# Supplementary material for: Immobilization of Peroxo-Heteropoly Compound and Palladium on Hydroxyapatite for the Epoxidation of Propylene by Molecular Oxygen in Methanol
Source: Molecules. 2022 Dec 20;28(1):24. doi: 10.3390/molecules28010024 (PMC9821923; doi:10.3390/molecules28010024)
Supplement: Supplementary file 1 [file molecules-28-00024-s001.zip › molecules-2033756-supplementary.pdf]

Table S1. Results of elemental analyses of Pd-PW-HAP before reaction by ICP and EDS.

Method	Chemical composition (wt.%)				Molar ratio of Ca/P
	Ca	P	W	Pd	
ICP[1]	26.1	15.2	13.2	1.2	1.33
EDS[2]	25.8	15.0	14.2	1.3	1.33
	25.6	14.9	14.5	1.4	1.33
	25.7	14.9	14.4	1.4	1.34

[1] ICP: inductively coupled plasma for elemental analyses.
[2] EDS: energy dispersive X-ray spectroscopy, analyzed 3 points on the Pd-PW-HAP particle in the TEM image of Pd-PW-HAP before reaction (Figure 6A).

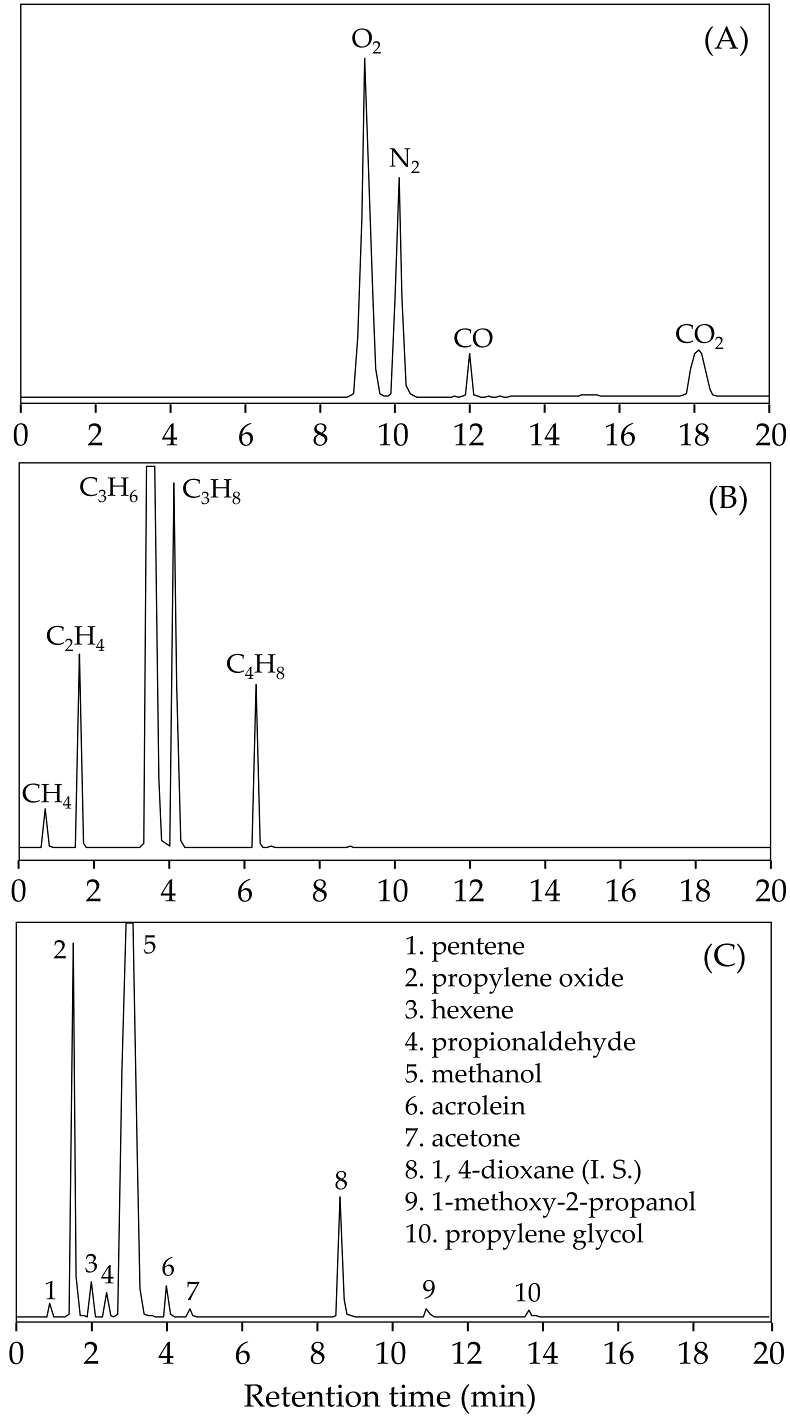

Figure S1. GC charts of products after reaction at 363 K for 8 h over Pd-PW-HAP. (A): TCD chart of gas products (column: Shincarbon-ST, 6 m × 3 mm; oven temperature: 313 K (11 min hold) → 448 K (rate: 15 K/min); (B) FID chart of gas products (column: RT-QPLOT, 30 m × 0.53 mm; oven temperature: 333 K (6 min hold) → 473 K (rate: 10 K/min); (C): FID chart of liquid products (column: PoraPLOT U, 30 m × 0.53 mm; oven temperature: 383 K (5 min hold) → 463 K (rate:10 K/min) → hold at 463 K).

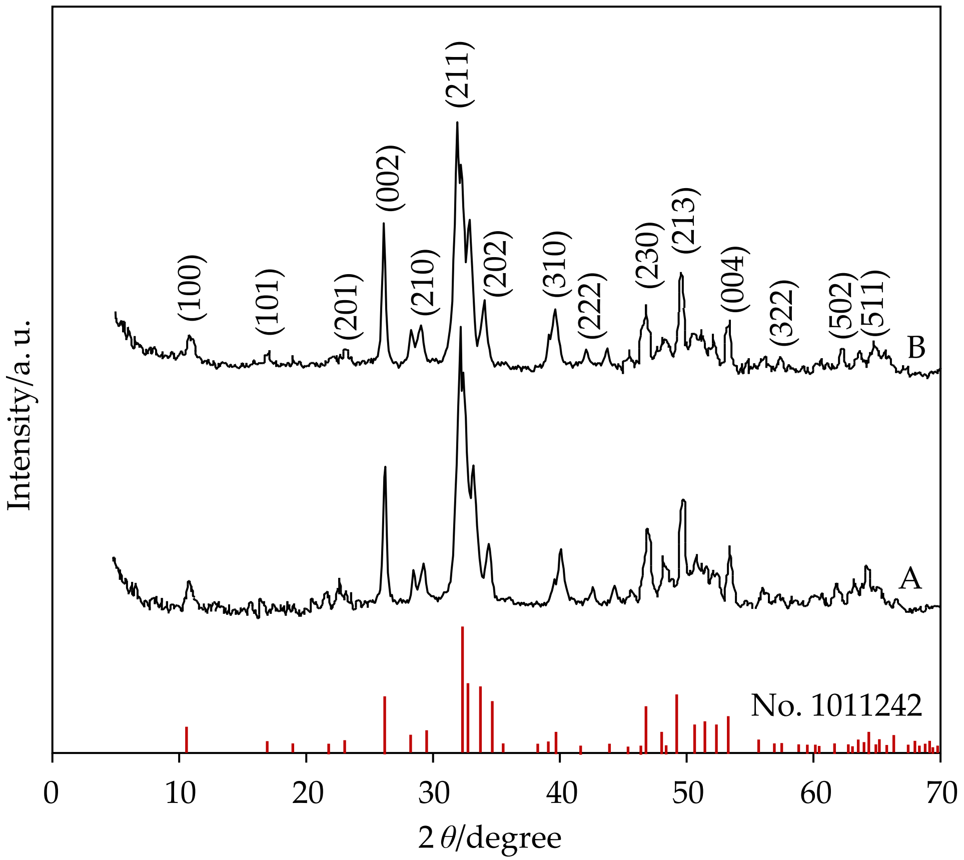

Figure S2. XRD patterns of HAP and Pd-PW-HAP before reaction. (A): HAP; (B): Pd-PW-HAP. Vertical bars in the lower graph correspond to the HAP phase from the Rigaku PDXL2 database (No 1011242)
